# Immunologically effective dose: a practical model for immuno-radiotherapy

**DOI:** 10.18632/oncotarget.25746

**Published:** 2018-08-07

**Authors:** Raphaël Serre, Fabrice Barlesi, Xavier Muracciole, Dominique Barbolosi

**Affiliations:** ^1^ Simulation & Modelling Adaptive Response for Therapeutics in Cancer (SMARTc), Center for Research on Cancer of Marseille, CRCM Inserm UMR1068, CNRS UMR7258, Aix Marseille Université U105, Institut Paoli Calmettes, Marseille, France; ^2^ Multidisciplinary Oncology & Therapeutic Innovations Department, Assistance Publique-Hopitaux Marseille, Marseille, France; ^3^ Department of Radiotherapy, Oncology, CHU La Timone, Assistance Publique-Hopitaux de Marseille, Marseille, France

**Keywords:** immunotherapy, radiotherapy, combination, fractionation, linear-quadratic model

## Abstract

**Objectives:**

Concomitant radiotherapy with immune checkpoint blockade could be synergistic. Out-of-field effects could improve survival by slowing or blocking metastatic spreading. However, not much is known about the optimal size per fraction and inter-fraction time in that new context.

**Methods:**

The new concept of Immunologically Effective Dose (IED) is proposed: it models an intrinsic immunogenicity of radiotherapy schedules, i.e. the fraction of immunogenicity that results from the choice of the dosing regimen. The IED is defined as the single dose, given in infinitely low dose rate, that produces the same amount of abscopal response as the radiation schedule being considered. The IED uses the classic parameters of the BED formula and adds two parameters for immunogenicity that describe the local availability of immune effectors within the tumor micro-environment. Fundamentally, the IED adds a time dimension in the BED formula and describes an intrinsic immunogenicity level for radiotherapy.

**Results:**

The IED is positively related to the intensity of the out-of-field, radiotherapy-mediated, immune effects described in some preclinical data. Examples of numerical simulations are given for various schedules. A web-based calculator is freely available.

**Conclusions:**

Out-of-field effects of radiotherapy with immune checkpoint blockers might be better predicted and eventually, radiotherapy schedules with better local and systemic immunogenicity could be proposed.

**Advances in knowledge:**

A model for the intrinsic level of immunogenicity of radiotherapy schedules, referred to as the Immunologically Effective Dose (IED), that is independent of the type of immunotherapy.

## INTRODUCTION

The abscopal effect of radiotherapy describes a rarely observed phenomenon in which one or more metastases located out of the irradiation field regress at some point in time after radiotherapy [1]. Described more than sixty years ago, the effect was rare, unpredictable and for these reasons, considered more like an interesting curiosity than an actual therapeutic target. Today it is largely suspected that a significant part of out-of-field effects could be mediated by the immune system, hence the recent introduction and large diffusion of immune checkpoint blockade has strongly revived interest for the subject, with promising clinical case reports [2], [3] a sizeable amount of preclinical results [4] and the exciting on-going development of a new hybrid discipline: immuno-radiotherapy.

The out-of-field mechanism could initially involve an in-field recruitment of dendritic cells (DC) and other types of Antigen Presenting Cells (APC) in the tumour micro-environment, attracted by radiation-induced inflammation and necrosis. However, these immune effectors have a strong radio-sensitivity: lymphocytes are among the most radiosensitive cells within the body (with doses as low as 0.5 Gy already strongly cytotoxic [5]), while DC and APC may survive higher doses but quickly suffer loss of function [6]. Hence, the local availability of immune effectors could be highly impacted by the choice of interfraction time. Meanwhile, the quantity of tumour antigens could be mainly driven by the size of the dose per fraction and the radio sensitivity of tumour cells: higher doses per fraction could release larger quantities of tumour antigens and produce an increased diversity of epitopes. This reasoning, supported by experimental confirmations from the preclinical setup [8] and several clinical reports, has aroused interest in hypo-fractionated schedules for recent clinical trials of radio-immunotherapy [17].

So far, only a few published mathematical models have focused on the equilibrium between the immune-stimulation and the immune-suppression induced by radiation [5], [9]. To our knowledge, no model explores specifically the relationship between fractionation and abscopal effect.

The biological assumptions underlying the IED model are partly like those previsously published in [9] by our team, which covered specific immune checkpoint blockers (such as anti-PD1 and anti-CTLA4); however, the present model focuses on the *intrinsic* part of immunogenicity, i.e. the part coming only from the radiotherapy schedule.

Other contributing factors to the abscopal response certainly exist: type of immunotherapy, relative scheduling, general state of immunosuppression of the patient, tumor size, local state of immunosuppression within the tumor microenvironment, possibly locations of irradiated site and abscopal target [10], p53 somatic mutations [13], etc. These factors should also be considered as part of a larger multivariate analysis, while the independent predictive value of the dosing regimen could be described by the IED.

## RESULTS

The IED model is compared to pre-clinical experiments of out-of-field responses for radiotherapy schedules used in metastatic breast cancer, as described by Dewan & al in [8].

The importance of interfraction interval on the intrinsic immunogenicity of radiotherapy schedules is shown on a simple theoretical example with two equal doses.

Last, values of intrinsic immunogenicity are computed for three radiotherapy schedules: the classic normofractionated schedule and two so-called “hybrid” schedules.

### IED is related to abscopal response (whereas BED or EQD2 are not)

In [8], Dewan & al. have injected TSA mouse breast carcinoma into syngeneic mice at two different sites. They irradiated one of the site with either 8 Gy x 3 fractions or 6 Gy x 5 fractions once daily, with and without an anti-CTLA4 antibody and they have measured the growth of the (non-irradiated) secondary site.

With combined anti-CTLA4 treatment, the average delays in growth with respect to control were: 6 days for 6 Gy x 5 fractions, 10 days for 8 Gy x 3 fractions. Hence, while these two schedules had almost identical BED and EQD2 (the definition of EQD2 is given below), the average growth delay of the 3 x 8 Gy schedule was 166% of that of the 5 x 6 Gy schedule. Since these two schedules have almost identical EQD2 and BED, the difference in the average out-of-field response, if it is not caused by randomness, can’t be explained by classic radiobiology models alone.

Interestingly, the IED Efficacy of 3 x 8 Gy *is more than twice the IED Efficacy of the* 5 x 6 Gy: apparently, it seems strongly associated with the intensity of the out-of-field response. Numerical values are shown in Table 1.

**Table 1:** IED efficacy, other dosing metrics and experimental correlates for results in [8].

RT schedule	α	α / β	EQD2 (Gy)	BED (Gy)	IED efficacy	out-of-field growth delay (days)
3 × 8 Gy (J1-J2-J3)	0.15	4	48	72	**74%**	**10**
5 × 6 Gy (J1 to J5)	0.15	4	50	75	32%	6

Remark 1: this association persists when using a more radiosensitive tumour with α = 0.35 or with varying values of α / β, or with different values of T_D_ (2 days, 3 days, 5 days) and T_IR_ (5 days, 7 days, 10 days). This can be verified with the online IED calculator: www.smartcalculators.online/ied.

Remark 2: the definition of EQD2 is standard, i.e. it is the total dose in Gy, that would produce the same cell kill as the schedule of interest, if it was administered in 2 Gy fractions (assuming a linear-quadratic model for cell kill).

### Impact of the interfraction time on IED Efficacy

**Figure 1:**
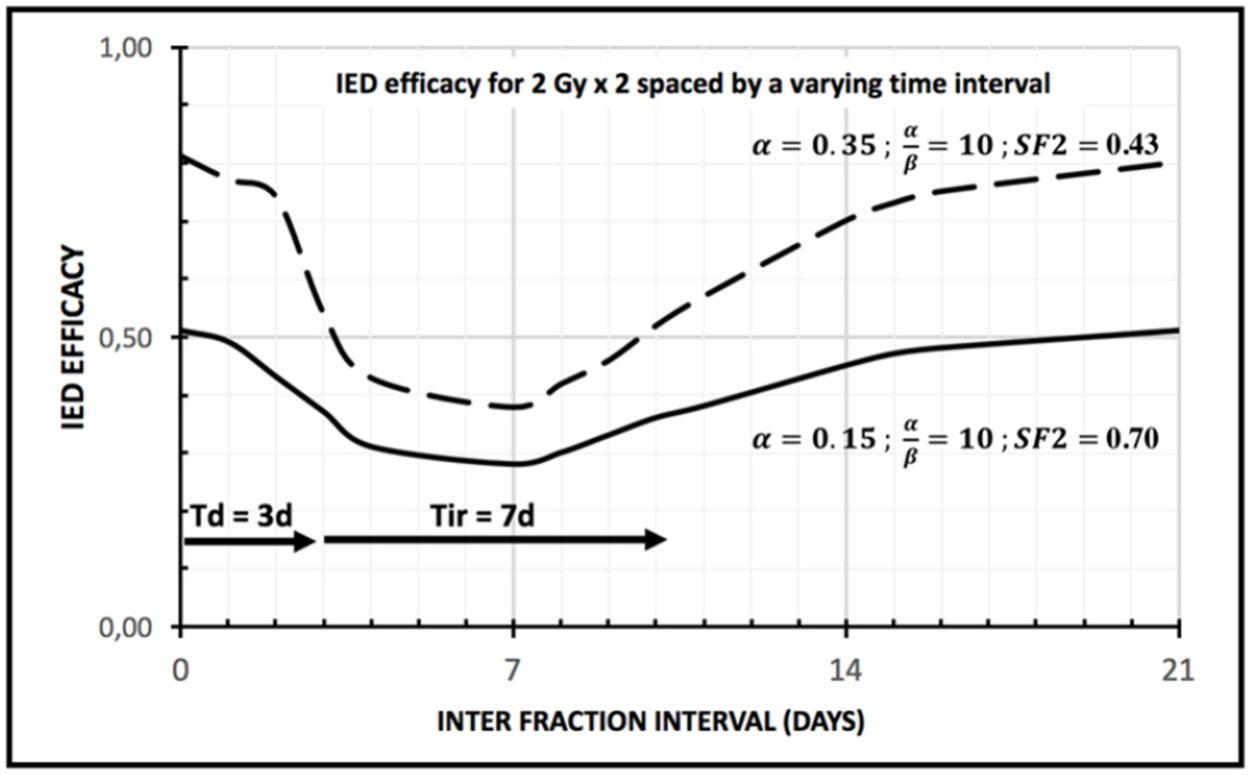
IED efficacy (between 0 and 100%) for two equal doses of 2 Gy, given with varying interfraction time (from 6h to 21 days); dashed line: highly radiosensitive tumour (Surviving Fraction 2 Gy = 0.43); solid line : intermediate radiosensitive tumour (Surviving Fraction 2 Gy = 0.70) ; the IED efficacy drops markedly if interfraction time T > Time to Death (T > T_D_) ; the IED efficacy recovers most of its value if the interfraction time T > sum of Time to Death and Time to Immune Response (T > T_D_ + T_IR_ ); the general shape is a steep drop in IED efficacy followed by a shallower recovery; notice how the impact of interfraction time decreases with radio resistance: strongly radio-resistant tumours should be less sensitive to the choice of the interfraction time

The interfraction time is a key factor for the local availability of intra-tumour immune effectors; with the IED model, this availability is increased either with a short inter-fraction time (< T_D_) or with long inter-fraction time (> T_D_ + T_IR_), but this availability is sharply decreased for intermediary inter-fraction values. This is illustrated on Figure 1 where two equal doses of 2 Gy are given with varying time interval, from 6 hours to 21 days. For the second radiation dose to not kill the immune effectors recruited by the necrotic tumour cells from the first radiation dose, the second radiation dose must be administered either very early (before necrosis) or very late (after completion of most of the immune stimulation). This has practical consequences if one wants to design more immunogenic radiotherapy schedules, as shown in the next section.

### IED Efficacy of various radiotherapy schedules

**Figure 2:**
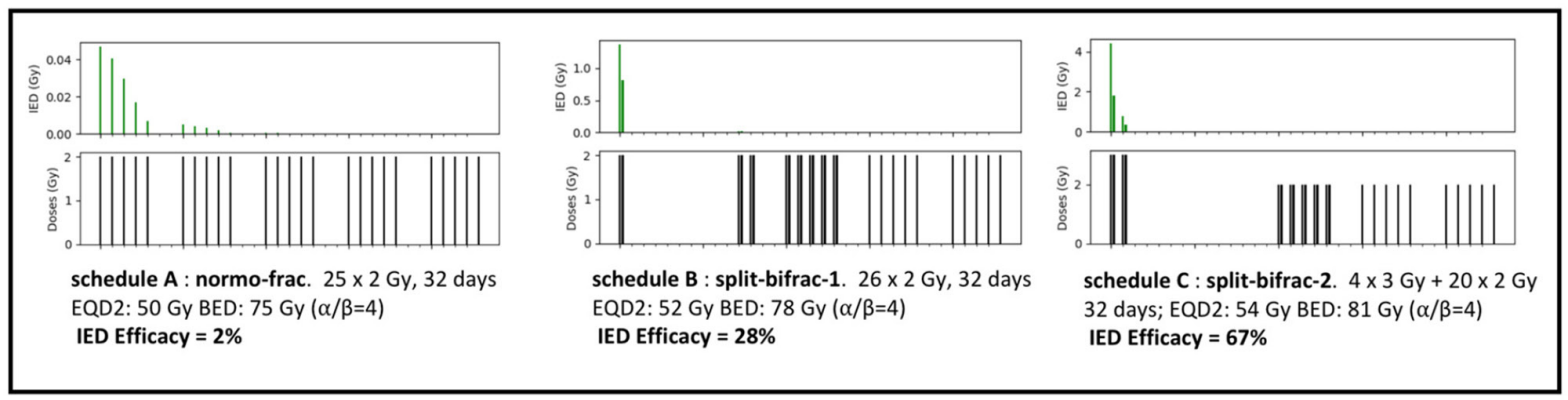
IED efficacy for three schedules: (A) normofractionated schedule (50 Gy in 25 fractions) very low IED Efficacy = 2%, (B) split-bifrac-1; proposal of new schedule that starts with one short bifractionation (2+2) for one day, split with 10-day break, 9 days of bi-fractionation (2+2) then 2 weeks of normofractionated, with same duration than A, same repopulation risk, EQD2 and BED than A, higher IED Efficacy = 28%; (C) split-bifrac-2 proposal of new schedule, starting with one short bifractionation (3+3) over 2 days, split with 13-day break, one week bifractionation (2+2), two weeks normofractionated, same duration than A, same repopulation risk, slightly higher EQD2 and BED than A, but much higher IED Efficacy = 67%

To demonstrate how the IED Efficacy indicator might be used, three schedules are provided: a classic normo-fractionated schedule in 2 Gy per fraction over 5 weeks that serves as a reference, and two new schedules, which are hybrid between normofractionated, split-course, and bi-fractionated (Figure 2). Despite the use of one break (10 and 13 days), the new schedules extend over the same total duration (32 days); they also have similar EQD2 and BED. Hence these new schedules should have similar tumor control probability and identical repopulation risk. The doses per fraction are also either identical (2 Gy) or slightly above (3 Gy) but then, only for a limited period (48h). The use of bi-fractionation is proposed to make up for the time lost during the break, but just for a minimum period and the last two weeks are administered in normo-fractionated fashion.

The IED model predicts a low intrinsic immunogenicity for the normo-fractionated schedule (IED Efficacy: 2%), and greater values for the two hybrid schedules (28% and 67%). These values can be obtained by using the online calculator at www.smartcalculators.online/ied.

## DISCUSSION

The clear positive relationship between IED Efficacy and the intensity of the out-of-field response observed with the data published in [8] is a strength of the model, but this needs to be confirmed on more experimental data.

The model prediction that normofractionated schedules should have lower immunogenicity than shorter schedules with higher doses per fraction seems coherent with some pre-clinical data [7], [8]. However, since out-of-field effects have also been described with normofractionated schedules [14], [15] more investigation is needed to clarify if this translates into a significant reduction of out-of-field responses.

The impact of interfraction time is consistent with pre-clinical data that have explored the dynamics of immune-infiltration in the tumor micro-environment after irradiation, such as Frey & al. [12]: with a peak of immune infiltration between 4 to 8 days after irradiation, radiation doses administered around that time range could be detrimental to anti-tumour immune response: this is indeed described by the IED model. Along a similar line, Formenti & al. [1] consider that the *local* availability of immune effectors inside the tumor micro-environment should be the main driver of anti-tumour immunity for immuno-radiotherapy, which is an idea at the core of the mechanistic approach of this model.

The IED model uses only two new “time” parameters T_D_ and T_IR_, whose signification is clear, since they control the speed of post-radiotherapy necrosis (T_D_) and the speed of immune infiltration (T_IR_) within the TME. Furthermore, thanks to a free access through an online calculator, there is no need to understand the fine details of the formula or to use a mathematical software.

The choice T_D_ = 3 days is subject to caution, even though its order of magnitude is in line with some pre-clinical experiments [16], but this needs to be further explored: one could investigate whether T_D_ could be also dose- or tumour-dependent or if, and when, T_D_ might differ from 3 days by a large amount.

For example, if the death of tumor cell was strongly delayed after irradiation (= very high T_D_), to such an extent that most of tumor death would occur long after the completion of the irradiation schedule, then the timing of radiation doses would not have any impact on immunogenicity (because all integrals **θ**_**ij**_ would be zero for j < N, and all **θ**_**iN**_would be one; hence, the matrix **Θ** would be constant). It would be interesting to explore the potential circumstances under which this case might occur.

The two examples of new “hybrid” schedules that might be more immunogenic than the classic normofractionated schedule are hypothesis-generating. These examples have been provided to show some potential directions for future research, while on the short term, more model validation is necessary.

Obviously, the differences in immunogenicity reflect the hypothesis on the model parameters, especially on T_D_ (and to a lesser extent on T_IR_): as discussed, larger values of T_D_ would reduce these differences.

The model proposes several hypotheses that could be experimentally challenged, for example:

• immunogenicity is not (only) about the dose per fraction, since large changes in theoretical immunogenicity could be obtained with identical dose per fraction, by “stacking” doses over shorter periods;
• the role of the interfraction time (which is inexistent in the classic linear-quadratic model) seems important for the local availability of immune effectors; however, this effect could be more important towards the start of the irradiation schedule (when live tumor mass is large) rather than towards its end (when live tumor mass should be much smaller);
• it follows that the total duration of the radiotherapy schedule does not have to be increased to improve immunogenicity, in contrast with the traditional split-course schedules which used to extend the total duration; this property is interesting since it avoids an increase in repopulation risk that could result from treatment extension;

The validity of the IED model for doses per fraction greater than 10 Gy is questionable: first, it relies on a linear-quadratic model whose validity above 10 Gy is not firmly established; second and more importantly, some pre-clinical data ([7], [8]) show that for very large doses per fraction (> 15 Gy), the IED model presented here would vastly overstate immunogenicity. However, an ad-hoc downward adjustment in the IED formula may provide a good correlate with [7], [8], even for > 10 Gy; this optional adjustment is available in the online calculator.

Finally, it is undeniable that predicting abscopal response is a difficult task, since it should depend on many parameters that may be patient-, organ-, tumour- or immunotherapy-specific. The IED model should not be misinterpreted as a tentative to replace this multi-dimensional problem by the computation of just one indicator. To give an obvious example, the radiotherapist understands that irradiating a 20g-tumour volume should produce more immune stimulation than the irradiation of a 2g-tumour, all other things being equal. A long list of other confounding factors could be made, whose influence is not in the scope of the proposed model, such as the immune status of the underlying tumor micro-environment (“hot” vs “cold” environment), the intrinsic efficacy of the concomitant immunotherapy, etc. Some of these factors might be added as covariates in the model, especially the state of the tumor micro-environment that could be described with the value of the time parameter T_IR_: colder (*resp. hotter*) environment could be associated with higher (*resp. smaller*) values of T_IR_.

But even in its simplest form with fixed parameters and no covariate, the IED formula provides a quick rule-of-thumb to assess the relevance of fractionation (from the standpoint of immunogenicity) and, on occasion, avoid the choice of under-performing schedules.

## MATERIALS AND METHODS

### Description of IED model

In the model, the radiotherapy-mediated anti-tumour immune response is proportional to the fraction of tumour cells killed by irradiation, whose content has been processed by immune effectors before the next irradiation. Hence, depending on the interfraction time, the number of killed tumour cells that contribute to the immune response may be significantly smaller than the total cell kill predicted by the BED.

More precisely, each radiation dose kills a certain number of cells, with a surviving fraction described by the two parameters (α, β) of a classic linear-quadratic model [11]. Then, lethally wounded cells die in a gradual process and produce tumour antigens in the tumor micro environment.

**Figure 3:**
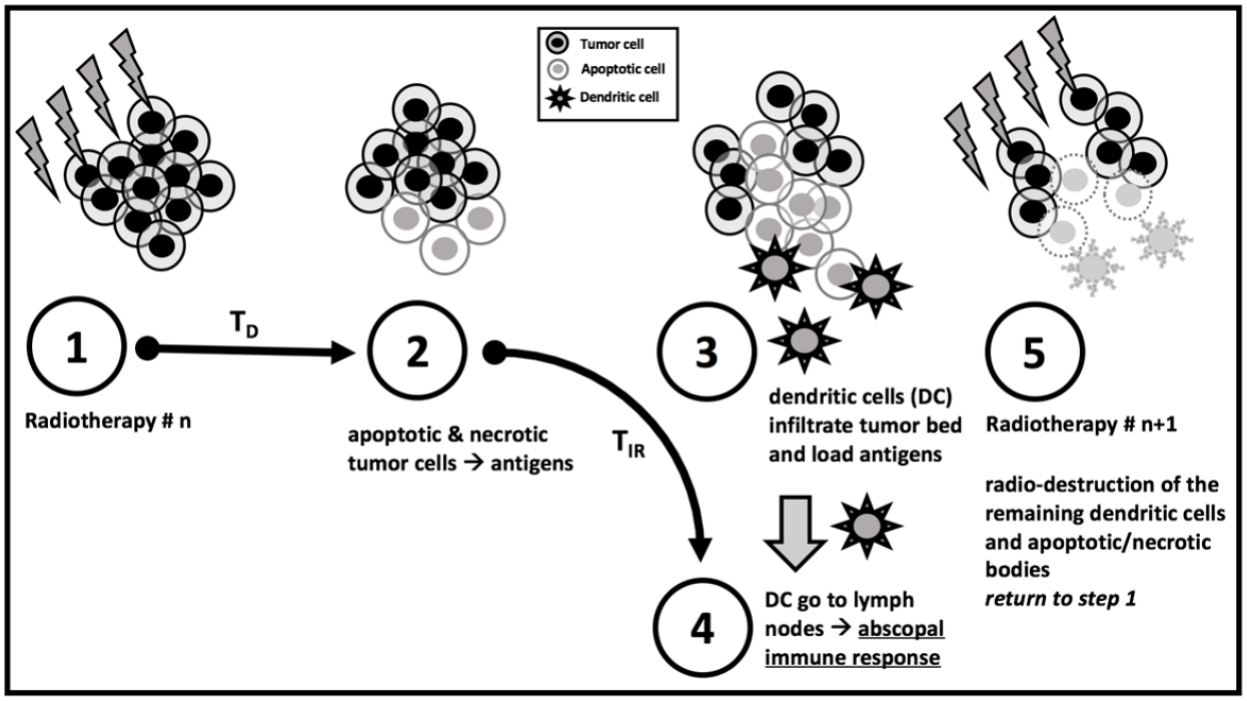
The 5-step dynamics of IED model Simplified graphical description. The actual model relies on a continuous process rather than a discrete step-by-step dynamic. **T**_**D**_ : Time to Death (days) **T**_**IR**_ : Time to Immune Response (days).

The time parameter T_D_ controls the speed of this antigen release: 50% of the tumour antigens are released after a time T_D_ (step 1 and 2 in Figure 3). Then, dendritic cells (DC) and other Antigen Presenting Cells (APC) take on tumour antigens and present them to lymphocytes in the draining lymph nodes. All numerical results were obtained with **T**_**D**_ = **3 days** (in coherence with data in [16], more details in the Discussion), while other T_D_ values could obviously be considered.

The time parameter T_IR_ (Time to Immune Response) controls the speed of this second process: 50% of completion is attained after a time T_IR_, following tumour antigen release (step 3 and 4 in Figure 1). Finally, the next radiation dose kills (or cause the dysfunction of) all immune effectors in the tumor micro-environment (step 5 in Figure 1). All numerical results here are obtained with **T**_**IR**_ = **7 days**, a reasonable estimate for such an immunologic phenomenon.

In summary, the model describes a succession of a progressive immuno-stimulation by dying tumour cells followed by an instantaneous immuno-suppression caused by the next irradiation.

For a *unique* dose, under the above assumptions, *all* killed tumour cells should contribute to the immune response. Hence, by defining IED as a unique dose given at infinitely low-dose rate, the IED value is obtained by solving the equation where the proportion of tumour cells that would be killed by this virtual dose, which is $1-e^{-\alpha \;\mathrm{ΙΕD}}$, equals the proportion of tumour cells that have contributed to the immune response as described above.

The direct solving of this equation yields a unique IED for any radiotherapy schedule, which is only defined by the dosing schedule and the choice of the radio-sensitivity parameters (α, β) and of the parameters that controls the immune response dynamics (T_D_, T_IR_). The IED is interpreted as a measure of the *intrinsic* contribution of the dosing schedule to immunogenicity.

The value $\text{E}=1-e^{-\alpha \;\mathrm{IED}}$ (a positive number < 1) is introduced to reflect better the efficacy of the radiotherapy schedule: 1 means maximal efficacy (100% of tumour volume contributed to immune response) and 0 means no immune efficacy (no volume contributed to immune response). This **E** value is named **IED Efficacy**.

### Mathematics of IED model

This section may be skipped by non-mathematicians. Using directly the model is possible by using the free web-based calculator, at www.smartcalculators.online/ied.

**Figure 4:**
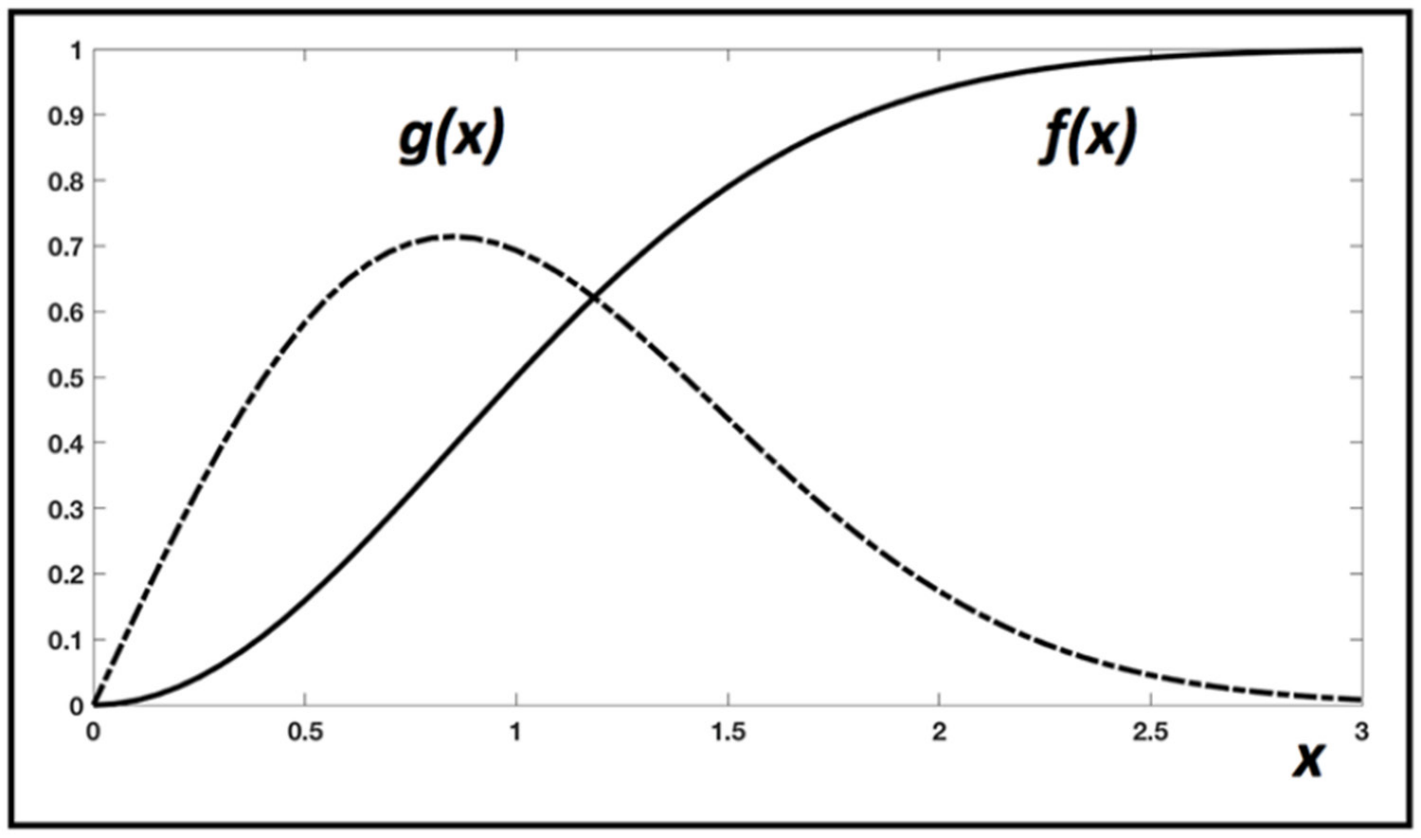
Graphical description of the two functions used in the IED model The number *g*(x) describes the gradual release of tumour antigens at time x, after irradiation at x = 0. The number *f*(x) describes the gradual immune stimulation, after tumour antigen release at x = 0. Here, x can be understood as a unit-less time variable, that is replaced by the ratio of an elapsed time over T_D_ (for *g*), or elapsed time over T_IR_ (for *f*). Their definitions are: $f\left(x\right)=1-{\left(\frac{1}{2}\right)}^{x^{2}}$ and $g\left(x\right)=\overset{\&}{f}\left(x\right)=2\;\ln\left(2\right)x{\left(\frac{1}{2}\right)}^{x^{2}}$, but other expressions could be used without changing the behaviour of the model, if the general shape of these curves is like this example.

With the above assumptions, for N equal radiation doses of D Gy given at times T_1_, T_2_…T_N_, the vector **E** of the quantities of “immunogenic” tumour antigens produced by each dose is equal to:

E=S Θ K

where:

**•** **S** is an N-by-N diagonal matrix with S_ii_ = (1-k_D_)^i-1^, the fraction of surviving tumour cells available just before the i^th^ radiation dose, where $k_{D}=1-e^{-\alpha D-\beta D^{2}}$, is the killed fraction for dose D;
**•** Θ is an N by N matrix where: $\theta_{\mathrm{ij}}=0$ for j<1, or $\theta_{\mathrm{ij}}=\int_{T_{j}}^{T_{j+1}}g\left(\frac{t-T_{i}}{T_{D}}\right)f\left(\frac{T_{j+1}-t}{T_{\mathrm{IR}}}\right)\mathrm{dt}$ for j≥i; the functions *f* and *g* are detailed in Figure 4;
**•** **K** is a N-by-1 vector whose components are equal to k_D_;

For example, with N = 3 doses, **E = S Θ K** is:

$$\left[\begin{array}{c} E_{1}\\ E_{2}\\ E_{3} \end{array}\right]=\left(\begin{array}{ccc} 1 & 0 & 0\\ 0 & 1-k_{D} & 0\\ 0 & 0 & {\left(1-k_{D}\right)}^{2} \end{array}\right)\left(\begin{array}{ccc} \theta_{11} & \theta_{12} & \theta_{13}\\ 0 & \theta_{22} & \theta_{23}\\ 0 & 0 & \theta_{33} \end{array}\right)\left[\begin{array}{c} k_{D}\\ k_{D}\\ k_{D} \end{array}\right]$$

Finally, IED is obtained by solving $E_{1}+\ldots +E_{N}=1-e^{-\mathrm{\alpha IED}}$.

The sum of the components of **E** is named **IED Efficacy**; it represents the % of tumor volume that is converted into “immunogenic” antigens, hence it is easier to interpret than the crude IED number.

The **θ**_**ij**_ coefficients are computed numerically (for j = N, the upper bound of the integral should be set to +∞).

The integral **θ**_**ij**_ represents the fraction of immunogenic antigens produced by tumour cells killed by the i^th^ radiation at time T_i_ but released later between the times T_j_ and T_j+1_; *g(x)* is the rate of release of tumour antigens per unit of time, after one irradiation (at x = 0); *f(x)* is the completion rate of the immune activation triggered by the release (at x = 0) of tumour antigens, hence it is a positive number smaller than one. The parameters T_D_ and T_IR_ control the speed of each phenomenon.

Hence, the matrix **Θ** depends only on the *timing* of radiation doses and on the time parameters (T_D_, T_IR_), whereas the vector **K** and the matrix S depend upon the *size* of the dose per fraction and the radio-sensitivity parameters (α, β).

From **E = S Θ K**, it is obvious that initial radiation doses contribute to the most part of immunogenic tumour antigens, because of the geometric decrease of the components of S.

## CONCLUSIONS

The Immunologically Effective Dose (IED) model complements the classic linear-quadratic formula by considering the dynamics of the immune infiltration within the tumor micro-environment. Then, it produces forecasts about the impacts of various radiotherapy schedules on the anti-tumor immune response.

Understanding the result of the IED model is easy with the IED Efficacy, which is the percentage of tumour volume that gives “immunogenic” tumour antigens: the closest to one, the better.

Obtaining the IED Efficacy of any radiotherapy schedule can be done with an online calculator that is freely available at www.smartcalculators.online/ied. Two parameters are needed: T_D_ and T_IR_, in addition to the classic parameters (α, β) of the linear-quadratic model.

It is not recommended to use the model with doses per fraction above 10 Gy, however, an ad-hoc adjustment parameter may be used if necessary.

With the IED algorithm, new radiotherapy schedules could be investigated in-silico, before being confirmed by preclinical data or clinical trials.

However, the validation of the model on more pre-clinical or clinical data is the next major step.
